# Mycotoxin Alternariol (AOH) Affects Viability and Motility of Mammary Breast Epithelial Cells

**DOI:** 10.3390/ijms22020696

**Published:** 2021-01-12

**Authors:** Karolina Kowalska, Dominika Ewa Habrowska-Górczyńska, Marta Justyna Kozieł, Kinga Anna Urbanek, Kamila Domińska, Agnieszka Wanda Piastowska-Ciesielska

**Affiliations:** 1 Medical University of Lodz, Department of Cell Cultures and Genomic Analysis, Zeligowskiego 7/9, 90-752 Lodz, Poland; dominika.habrowska@umed.lodz.pl (D.E.H.-G.); marta.koziel@umed.lodz.pl (M.J.K.); kinga.urbanek@umed.lodz.pl (K.A.U.); 2 Medical University of Lodz, Department of Comparative Endocrinology, Zeligowskiego 7/9, 90-752 Lodz, Poland; kamila.dominska@umed.lodz.pl

**Keywords:** alternariol, mycotoxins, oxidative stress, motility, mammary gland

## Abstract

Mycotoxins are present in everyday diet as common food and feed pollutants. A part of them is still concerned as so-called emerging mycotoxins. Due to the lack of toxicity data, the safety limits and detail molecular mechanism have been not established yet for all of them. Alternariol (AOH), as one of these mycotoxins, produced by *Alternaria* species, is so far reported as an estrogenic, genotoxic, and immunomodulatory agent; however, its direct effect on human health is not known. Especially, in the case of hormone-dependent tissues which are sensitive to both endogenic, as well as external estrogenic agents, it might be crucial to assess the effect of AOH. Thus, this study evaluated how exposure to AOH affects viability and motility of the human normal mammary gland epithelial in vitro model. We observed that AOH significantly affects viability of cells in a time- and dose-dependent manner. Moreover, the induction of oxidative stress, DNA damage, and cell cycle arrest in the G2/M cell cycle phase was observed. The motility of 184A1 cells was also significantly affected. On the molecular level, AOH induced antioxidative stress response via activation of Nuclear factor erythroid 2-related factor 2 (NRF2) signaling pathway agents, as well as decrease in the phosphorylation of protein kinase B (Akt) and p44/42 (ERK 1-2) molecules, indicating that AOH might affect crucial signaling pathways in both physiological and pathophysiological processes in breast tissue.

## 1. Introduction

Contrary to most of the endothelial cells, the mammary gland develops postnatal and includes complex epithelial remodeling in response to steroid hormones and growth factors. The steroid hormones mainly responsible for mammary gland remodeling are estrogens and progesterone [1]. A specific group of both natural, as well as synthetic, substances present in every day food and feed may interfere with the hormonal system and affects both hormonal balance, as well as hormone-dependent tissues, directly. These “endocrine disruptors” (EDC), which mimic natural estrogens, are now a group of more than 450 compounds [2] which significantly affects hormonal balance in humans and constitutes a global health problem [3]. Estrogens, both endogenous and xenoestrogens, as well as phytoestrogens, play an important role both in the development of breast epithelium by stimulating proliferation and ductal morphogenesis, as well as breast carcinogenesis [4]. Moreover, the growing number of breast cancer cases in young women indicates that environmental factors might participate in breast carcinogenesis [5]. However, the effect of a naturally-occurring EDC in food on human health is not fully elucidated.

Mycotoxins-toxic secondary metabolites of fungi are common pollutants in feed and food [6]. Mycotoxin alternariol (AOH), a toxin produced by *Alternaria* fungi is considered as an estrogenic mycotoxin [7]; however, its direct impact on human health has not been established yet [8]. AOH is found in fresh and processed fruit, vegetables, nuts, and grain [9]. Due to low number of toxicological and occurrence studies, AOH is considered as emerging mycotoxin, and no safety daily limits have been established by European Food Safety Authority (EFSA) [10]. So far, AOH has been reported to be genotoxic [11], act as an immunomodulatory agent [12], and possess mutagenic and carcinogenic potential. The association between AOH exposure and esophageal cancer was also suggested [13]. Besides its estrogenic activity, modulation of proliferation, and cell cycle progression in estrogen-responsive Ishikawa cells [14], AOH was reported to cause cell cycle arrest and oxidative stress in Caco-2 cells [15] and inflammation response via nuclear factor-kappa B (NFκB) signaling pathway [16]. It is suggested that AOH-induced DNA damage is associated with oxidative stress and interaction with DNA topoisomerases. AOH is reported to act as topoisomerase I and II inhibitor and, in consequence, induce double strand breaks (DSB) of DNA [10].

Generation of reactive oxygen species (ROS), as a part of oxidative stress in cells, plays a crucial role both in physiology, as well as pathophysiology; thus, the balance between ROS production and detoxication is especially crucial. ROS overproduction in cells is considered as one of the causative factors of the development of cancer [17]. Moreover, the estrogen-induced ROS generation is suggested to contribute to breast cancer via modulation of DNA synthesis, phosphorylation of kinases, and activation of transcription factors, e.g., NFκB and nuclear factor erythroid 2-related factor 1 (NRF1) [18]. Previous studies showed that AOH might generate oxidative stress in colon cancer cell line Caco-2 [15], HT-29 [19], and KYSE510 esophageal cells [20]; however, the effect on breast cells is not known. It is highly possible that AOH might induce oxidative stress in normal breast epithelial cells and participate with other genotoxic and mutagenic agents in carcinogenesis. Thus, we decided to evaluate if AOH induces oxidative stress in normal breast epithelial cells, as well as its effect on the motility of cells.

## 2. Results

### 2.1. AOH Decreases Viability of Normal Mammary Gland Epithelial Cells and Changes Their Morphology

**Figure 1:**
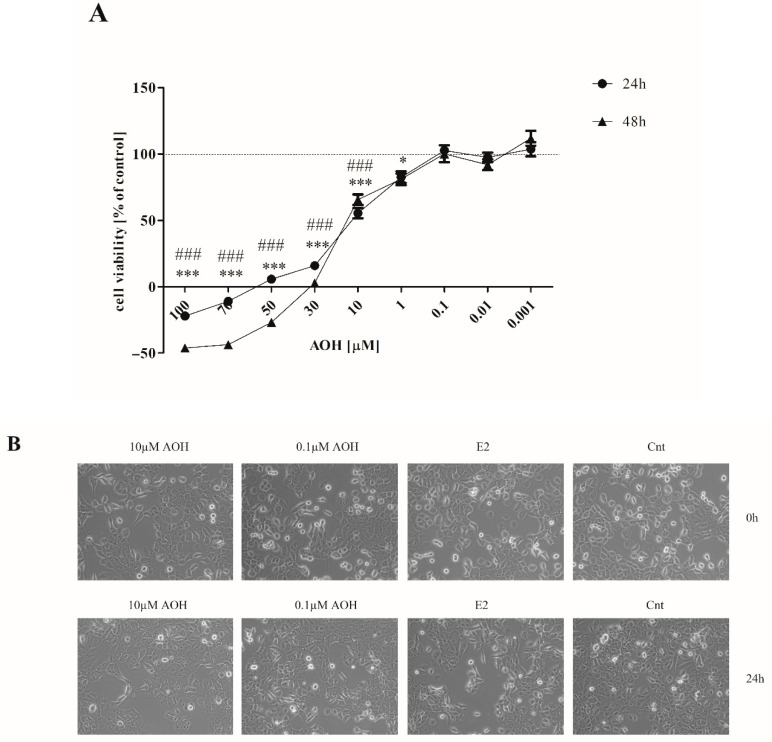
The effect of alternariol (AOH) on viability and morphology of mammary gland epithelial 184A1 cells. (**A**) The viability of cells was evaluated with AlamarBlue reagent for 24 and 48 h. The results are expressed as % of control and presented as mean ± SE. One-way ANOVA was used to calculate statistically significance. *P* lower than 0.05 was considered as statistically significant. * *p* < 0.05, *** *p* < 0.001 as compared to control for 24-h results, ### *p* < 0.001 as compared to control for 48 h exposition to AOH. (**B**) Changes in cells morphology observed after 24 h. Magnification 40×. AOH—alternariol, E2—10 nM estradiol, Cnt—control.

To evaluate the dose-response curve, we tested a broad range of AOH concentrations from very high (100 µM) to very low (0.001 µM) in two time points: 24 h and 48 h (Figure 1A). We observed that AOH significantly decreases viability of 184A1 cells in a dose- and time-dependent manner. The concentrations below 10 µM were sufficient to decrease the viability of cells significantly (***/### *p* < 0.001 for 24 and 48 h, respectively) as compared to the control cells. Based on the IC_50_ value (23.97 µM) calculated for 24-h time point, we decided to choose two concentrations of AOH for the rest of experiments: the one for which a significant decrease in cell viability was observed, however, higher than IC_50_ value (10 µM) and a lower dose (0.1 µM). We also observed that AOH in higher concentration induces changes in morphology of cells visible as round-shaped nuclei and a lesser number of dividing cells, as well as higher number of graininess in cells, not observed in the similar extend in a lower dose of AOH (Figure 1B).

### 2.2. AOH Induces Oxidative Stress, DNA Damage, and Cell Cycle Arrest in G2/M Cell Cycle Phase

**Figure 2:**
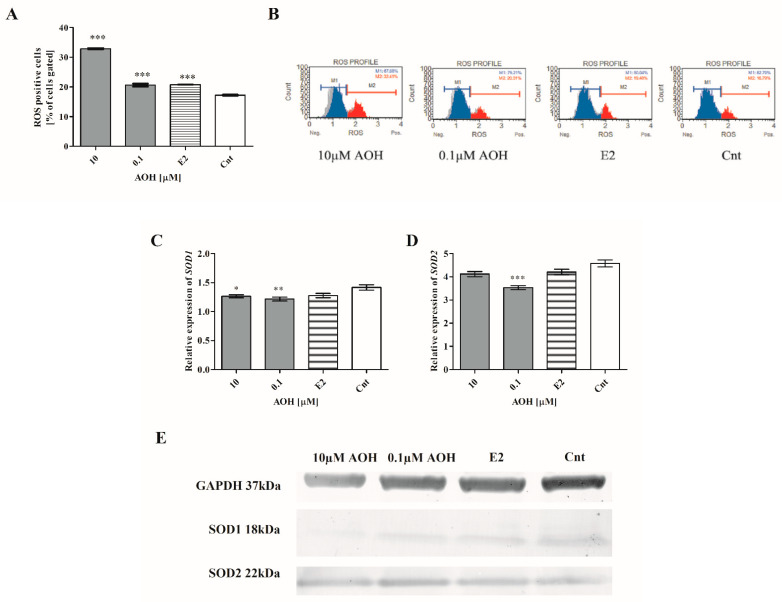
AOH induces oxidative stress in mammary gland epithelial cells. (**A**) ROS positive cells were counted with flow cytometry and expressed as % of gated cells. The results are presented as mean ± SE. (**C**,**D**) Relative expression of *SOD1* and *SOD2* evaluated with RTqPCR. One-way ANOVA was used to calculate statistically significance. *P* lower than 0.05 was considered as statistically significant. * *p* < 0.05, ** *p* < 0.001, *** *p* < 0.001 as compared to control. (**B**) Representative results of flow cytometry results. (**E**) Representative results of Western blot. ROS–reactive oxygen species, AOH–alternariol, E2–10 nM estradiol, Cnt–control, SOD1–superoxide dismutase 1, SOD2–superoxide dismutase 2.

An induction of oxidative stress in cells is reported to be a cause of the exposure to mycotoxins [19]; thus, we evaluated if decreased viability of 184A1 cells is associated with production of ROS in cells. Firstly, we observed a statistically significant increase in the number of ROS positive cell (*** *p* < 0.001) as compared to the non-treated cells. An almost two-fold increase was observed for 10 µM of AOH; for lower concentration, a similar to estrogen (E2) increase was observed (Figure 2A,B). The increase in the number of ROS positive cells was associated with changes in the expression of genes associated with the response to oxidative stress. A decrease in the SOD-1 expression was observed for both doses of AOH, both at the gene (Figure 2C) and protein level (Figure 2E). A decrease in the expression of SOD-2 was also observed, significant for lower dose of AOH and not significant for higher dose (Figure 2D,E).

**Figure 3:**
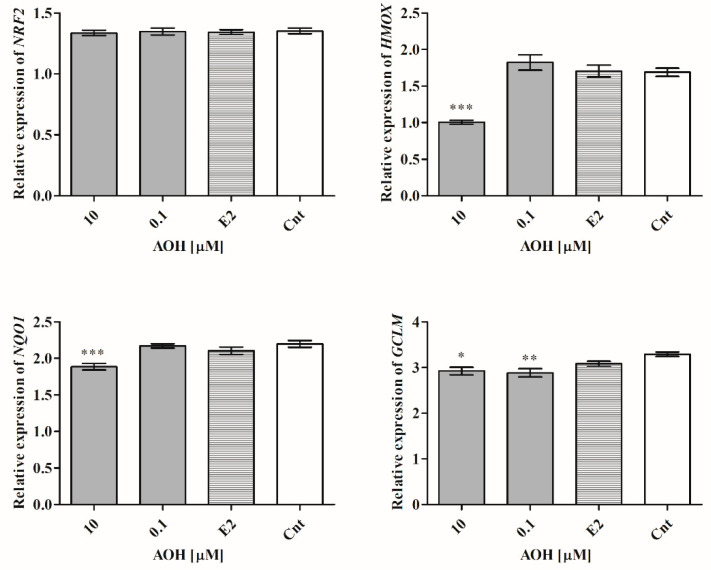
AOH modulates expression of *HMOX1*, *NQO1*, and *GCLM*, but not *NRF2*. The results of relative expression were obtained in RTqPCR and expressed as mean ± SE. One-way ANOVA was used to calculate statistically significance. *P* lower than 0.05 was considered as statistically significant, * *p* < 0.05, ** *p* < 0.01, *** *p* < 0.001 as compared to control. AOH–alternariol, E2–10 nM estradiol, Cnt–control, *HMOX*–heme oxygenase 1, *NQO1*–NADPH quinone dehydrogenase 1, *GCLM*–glutamate-cysteine ligase regulatory subunit.

Next, the expression of Nrf2 signaling pathway was evaluated, as a main responsive element to oxidative stress in cells. Although the expression of *NRF2* was almost not changed (Figure 3), the expression of heme oxygenase 1 (*HMOX1*), NADPH quinone dehydrogenase 1 (*NQO1*), and *GCLM* was significantly decreased after treatment with 10 µM of AOH (*** *p* < 0.001, *** *p* < 0.001, * *p* < 0.05), and 0.1 µM of AOH in the case of glutamate-cysteine ligase regulatory subunit (*GCLM*) gene (** *p* < 0.01), as compared to control.

**Figure 4:**
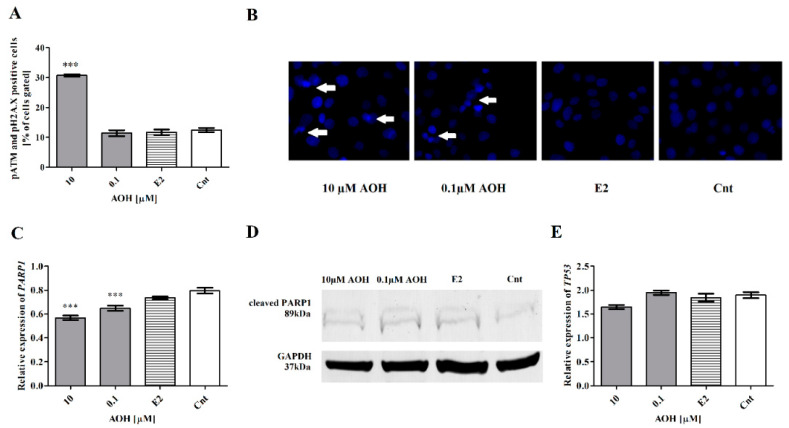
AOH induces DNA damage in mammary gland epithelial cells. (**A**) DNA damage was detected with Muse Multicolor DNA Damage Kit. The results are expressed as mean ± SE. (**B**) 2-(4-Amidinophenyl)-6-indolecarbamidine dihydrochloride (DAPI) staining of cells; white arrows present cells with changed morphology of nuclei. (**C**) The relative expression of *PARP1* gene obtained in RTqPCR expressed as mean ± SE. (**D**) The results of Western blot. (**E**) The relative expression of *TP53* gene from RTqPCR results. The results are expressed as mean ± SE. One-way ANOVA was used to calculate statistically significance. *P* lower than 0.05 was considered as statistically significant, *** *p* < 0.001 as compared to control. AOH–alternariol, E2–10 nM estradiol, Cnt–control, PARP1–Poly (ADP-ribose) polymerase 1, TP53–transformation related protein 53.

The induction of oxidative stress in cells might be associated with DNA damage [21]. To evaluate if AOH might induce oxidative stress in mammary gland epithelial cells, the cells expressing phospho-ataxia telangiectasia mutated kinase (p-ATM) and phospho-histone H2A.X (pH2A.X) were stained and counted with flow cytometry. We observed that AOH in the dose of 10 µM significantly increased DNA damage in cells (*** *p* < 0.001) as compared to control cells (Figure 4A), whereas the lower dose of AOH caused almost no effect. We observed a changed morphology of nuclei: not uniformly stained with 2-(4-Amidinophenyl)-6-indolecarbamidine dihydrochloride (DAPI), not round-shaped in both doses of AOH, confirming previous results (Figure 4B). However, the changes in nuclei staining were also observed for lower dose of AOH and were not confirmed by flow cytometry. Moreover, we observed that AOH in a dose-dependent manner significantly reduces expression of poly(ADP-ribose) polymerase (*PARP1*)*,* a first responder in cells to DNA damage, as compared to control (*** *p* < 0.001) (Figure 4C). On the protein level, the decrease in the expression of PARP1 was detectable only for higher concentration of AOH (Figure 4D). We also observed a decreased expression of a regulator of DNA repair- transformation related protein P53 (*TP53*) after treatment with 10 µM of AOH (Figure 4E); however, no significant changes were observed.

**Figure 5:**
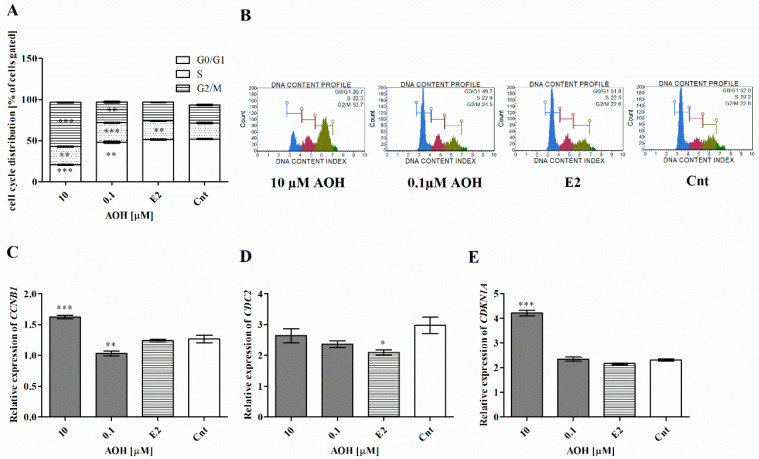
AOH modulates cell cycle progression in 184A1 cells. (**A**) Cell cycle progression after AOH treatment counted with Muse Cell Cycle Kit. (**B**) Representative results of the flow cytometry. (**C**–**E**) The relative expression of *CCNB1, CDC2*, and *CDKN1A* obtained in RTqPCR. The results are presented as mean ± SE. One-way ANOVA was used to calculate statistically significance. *P* lower than 0.05 was considered as statistically significant. * *p* < 0.05, ** *p* < 0.01, *** *p* < 0.001 as compared to control. AOH–alternariol, E2–10 nM estradiol, Cnt–control, *CCNB1*–cyclin B1, *CDC2*–cyclin-dependent kinase 1, *CDKN1A*–cyclin-dependent kinase inhibitor 1 (p21).

Cell cycle regulation and progression is a basic process in cells, and disturbances in it are present both during oxidative stress and carcinogenesis. Based on this assumption, we evaluated the cell cycle progression in cells treated with AOH and observed that AOH increases the number of cells in G2/M and S cell cycle phase (** *p* < 0.01 and *** *p* < 0.001) (Figure 5A,B). Simultaneously, the increase was associated with a significant decrease in the number of cells in G0/G1 (*** *p* < 0.01, ** *p* < 0.001, respectively, for 10 µM and 0.1 µM AOH). Moreover, we observed a significantly increased expression of cyclin B1 (*CCNB1*) for higher dose of AOH (*** *p* < 0.001) and contradictory effect for lower dose of AOH (** *p* < 0.01). Although we did not observe any significant changes in the expression of another regulator of G2/M cell cycle progression cyclin-dependent kinase 1 (*CDC2*), for the higher dose of AOH, a statistically significant increase in the expression of cyclin-dependent kinase inhibitor 1 (p21) (*CDKN1A*) (*** *p* < 0.001) was observed (Figure 5C–E).

### 2.3. AOH Modulates Motility of Mammary Gland Epithelial Cells

**Figure 6:**
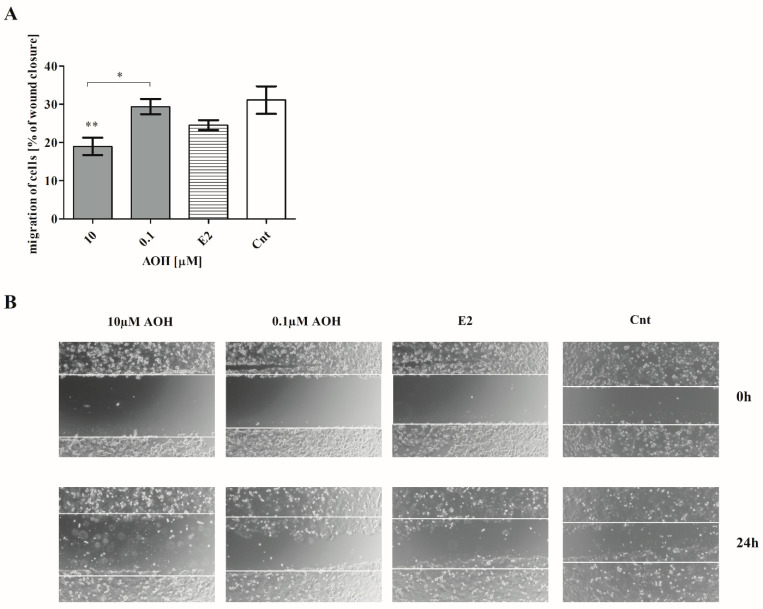
AOH decreases migration of 184A1 cells. (**A**) The results of wound healing assay are expressed as mean ± SE. One-way ANOVA with Bonferroni post hoc test was conducted to calculate statistical significance. *P* lower than 0.05 was considered as statistically significant. * *p* < 0.05, ** *p* < 0.01 as compared to control cells. (**B**) Representative results of wound healing assay. Images were obtained with Olympus microscope, magnitude 40×. AOH–alternariol, E2–10 nM estradiol, Cnt–control.

Cells motility is observed both during physiological and pathological processes, especially in carcinogenesis. It is associated with cells migration, invasion, adhesion, and interaction with extracellular matrix proteins (ECM). Especially, in the case of the mammary gland, reorganization of its structure plays a crucial role. Thus, we decided to evaluate if and how AOH affects cells motility. Firstly, we observed that AOH affect cells migration (Figure 6A,B). After 24 h treatment with AOH in a dose of 10 µM, a statistically significant decrease in the cells migration was observed, as compared to control cells (** *p* < 0.01). A similar effect, but in a lower extend, was observed for AOH in a dose of 0.1 µM (* *p* < 0.01).

**Figure 7:**
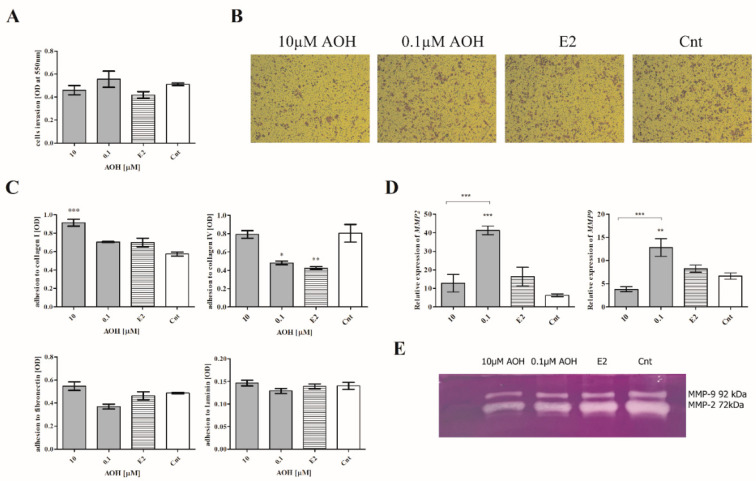
AOH modulates the adhesion of 184A1 cells. (**A**) The results of the cell culture inserts are expressed as mean ± SE. (**B**) Representative results of stained cells after invasion assay, magnitude 40×. (**C**) The results of cells adhesion to ECM proteins are expressed as mean ± SE. (**D**) RTqPCR was performed to analyze the expression of *MMP-2* and *MMP-9*. (**E**) zymography assay was performed to analyze the expression of *MMP-2* and *MMP-9*. The results are expressed as mean ± SE. One-way ANOVA was used to calculate the statistical significance, *p* < 0.05 was established as significant. * *p* < 0.05, ** *p* < 0.01, *** *p* < 0.001 as compared to control. AOH–alternariol, E2–10 nM estradiol, Cnt–control, ECM–extracellular matrix, *MMP-2*–metalloproteinase 2, *MMP-9*–metalloproteinase 9.

Next, the invasion and adhesion of cells, as well as the activity of metalloproteinases, was evaluated. We observed that AOH only slightly modulated the invasion of cells, and these changes were not significant (Figure 7A,B), whereas the adhesion of 184A1 cells was significantly modulated by AOH. We observed that 10 µM of AOH significantly increased the adhesion of cells to collagen I (*** *p* < 0.001) and not significantly in the case of collagen IV, laminin, and fibronectin (Figure 7C). Interestingly, the adhesion to ECM proteins was significantly decreased after treatment of cells with 0.1 µM AOH, similarly to estrogen (E2) treatment (collagen IV, * *p* < 0.05 and ** *p* < 0.01, respectively). The changes in cells migration and adhesion after high-dose AOH treatment were associated with the decrease in the expression and activity of metalloproteinase 2 (MMP-2) and 9 (MMP-9) evaluated with RTqPCR and zymography assay (Figure 7D,E). A contradictory effect was observed for lower dose of AOH: a statistically significant increase in the *MMP-2* and *MMP-9* expression was observed (*** *p* < 0.001, ** *p* < 0.01).

**Figure 8:**
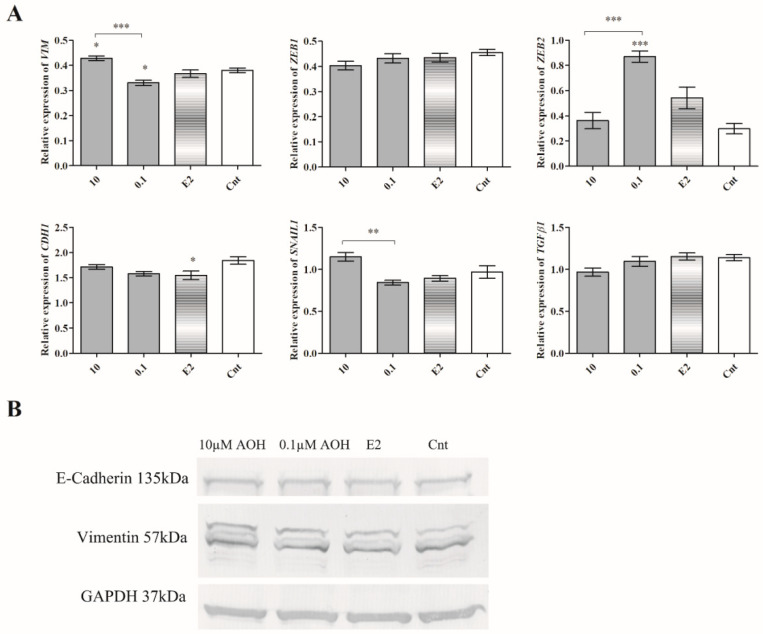
The expression of EMT regulators. (**A**) The results of RTqPCR are expressed as mean ± SE. One-way ANOVA was used to calculate the statistical significance, *p* < 0.05 was established as significant. * *p* < 0.05, ** *p* < 0.01, *** *p* < 0.001 as compared to control. (**B**) The representative results of Western blot. AOH–alternariol, E2–10 nM estradiol, Cnt–control, *CDH1*–E-cadherin, *VIM*–vimentin, *ZEB1*–Zinc finger E-box-binding homeobox 1, *ZEB2*–Zinc finger E-box-binding homeobox 2, *SNAIL1*–Zinc finger protein SNAI1.

Epithelial to mesenchymal transition (EMT) is both a physiological, as well as pathological, process that occurs in cancer cells [22]. It is associated with changed morphology of cells, as well as expression of so-called EMT markers: E-cadherin (CDH1) and vimentin (VIM), as well as transcription factors: Zinc finger E-box-binding homeobox 1 (ZEB1), Zinc finger E-box-binding homeobox 2 (ZEB2), Zinc finger protein SNAI1 (SNAIL1), and transforming growth factor β1 (TGFβ1). Due to observed changes in cells migration and adhesion, we decided to evaluate the expression of EMT markers. A contradictory effect between two doses of AOH was observed for *VIM*, *ZEB2*, and *SNAIL1* expression. We observed that 10 µM AOH significantly increased the expression of *VIM* (* *p* < 0.05), whereas lower dose of AOH 0.1 µM significantly decreased expression of *VIM* (* *p* < 0.05) and increased expression of *ZEB2* (*** *p* < 0.001). In the case of *ZEB1* and *TGFβ1*, we observed a dose-dependent decrease; however, it was not significant (Figure 8A). On the protein level, we did not observe the significant changes in the expression of *CDH1* and *VIM* (Figure 8B), indicating that the observed changes in the expression are not directly associated with EMT process.

**Figure 9:**
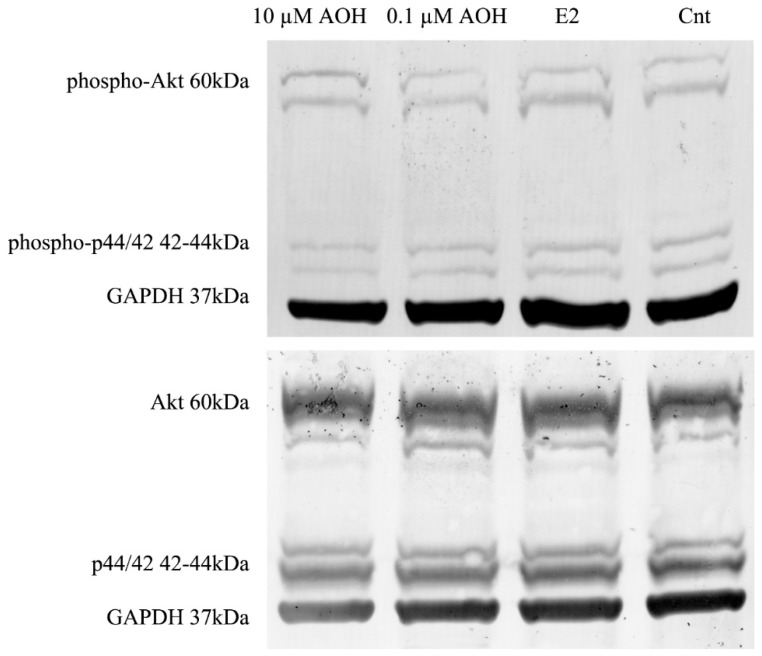
The expression of Akt and p44/42. Representative results of Western blot analysis. Akt–protein kinase B, p44/42–Erk1, AOH–alternariol, E2–estradiol 10 nM, Cnt–control.

We also observed that treating the cells with AOH decreased the expression of protein kinase B (Akt) and Erk1/2 (p44/42) and their phosphorylated forms (Figure 9). Phosphoinositide 3-kinase (PI3K)/Akt and mitogen-activated protein kinases (MAPK) signaling pathways are the key intracellular pathways in breast cancer responsible for the proliferation and metastases [23]. We observed that AOH decreased both expression of Akt and p44/42, as well as its phosphorylated forms (Table 1). The highest decrease in the expression was observed for higher dose of AOH, which confirms previously observed decrease in the cells viability.

## 3. Discussion

The evaluation and reports considering the molecular effect of the mycotoxins of human health are crucial to estimate and understand their effects both on human, as well as animal, health. The effect of *Alternaria* mycotoxins has been not fully elucidated yet. Thus, we and other research groups are focused on an elucidation of the mechanism of these mycotoxins both with in vitro and in vivo studies. Due to the fact that AOH is reported to be potentially an estrogenic [7], as well immunomodulatory [21], agent, it is possible that presence of this mycotoxin might affect the physiology of breast tissue which is known from its sensitivity to hormonal changes [18]. An elevated lifetime estrogens exposition, both endo- and exogenous, has been shown to be a major risk factor for hormone-sensitive organs [18]. Our study, for the first time, showed that AOH is able to affect viability and motility of normal mammary gland cells. We observed that AOH induces oxidative stress and DNA damage with changes in cell cycle regulation. On the molecular level, we observed that AOH triggers anti-oxidative stress response associated with HO-1 and Akt/Erk1 cell signaling pathways. Our results also showed, for the first time, that AOH significantly affects cells migration and adhesion. This effect was associated with regulation of MMPs activity.

Oxidative stress is one of the main processes involved in chronic diseases and cancers. The balance between oxidant and antioxidant agents is crucial: a physiological level of oxidative stress, on the one hand, is necessary for proper functioning of cells, whereas, on the other hand, elevated oxidant status induces damage of cells. AOH was previously reported to induce oxidative stress in human colon cancer cells HT-29, human liver cancer cells HepG2, and esophageal cancer cells KYSE510 [24]. We also observed that AOH induces oxidative stress in normal mammary gland cells 184A1 for both tested doses in a dose-dependent manner associated with changes of the expression of SOD1 and SOD2- the main antioxidative enzymes. Antioxidant response element (ARE) activation is controlled by the NRF2 signaling pathway. Previously, AOH was reported to increase the expression of *NRF2* and its response genes [19]; however, we did not observe a change in *NRF2* expression, but the expression of *HMOX1*, *NQO1*, and *GCLM* was significantly decreased. Due to the fact that Nrf2 itself is not able to bind to ARE, it creates a complex with small transcription factor Maf (sMAF) and then binds to ARE and activates the expression of detoxifying enzymes: *NQO1*, *HMOX1*, *SOD*, and *GCLM*. Due to the fact that AOH affects expression of all NRF2 response elements, we postulate that NRF2 signaling pathway might be a response of normal mammary gland epithelial cells to AOH-induced oxidative stress.

AOH was previously reported to decrease proliferation of cells by long lasting cell cycle arrest and, in consequence, the death of cells. It was postulated that cell cycle arrest in the G2 cell cycle phase might be the cause of DNA damage or the fact that AOH is reported to act as topoisomerase II poison [10,25]. In line with previous observation, we also observed that AOH induces DNA damage in cells and cell cycle arrest in the G2/M cell cycle phase. In addition, we evaluated the expression of *CCNB1*, *CDC2*-main regulator of G2/M transition, and *CDKN1A*-cell cycle regulator, as well as responsive element in to different stimuli to arrest cell cycle and ensure genomic stability [26]. We reported that observed DNA damage and cell cycle arrest are associated with the significantly increased expression of *CCNB1* and *CDKN1A*. Next, we evaluated the expression of *PARP1* and *TP53* as the sensors of DNA damage. PARP1 is reported to recognize DNA damage and enables its repair by recruiting the DNA repair proteins, thus playing a pivotal role in maintaining genomic stability [27]. We did not observe significant changes in p53 expression, indicating that observed decrease in cell viability and oxidative stress was not associated with apoptosis, which is in line with previous research indicating that AOH induces necrosis or autophagy in cells [28]. However, the expression of PARP1 both on the gene and protein level was significantly decreased after treatment of cells with 10 µM of AOH, which confirms observed oxidative stress and DNA damage caused by AOH.

The transformation of cells into cancer cells and their metastases is a complex and multistep process which involves formation of primarily tumor cells, their growth, EMT, migration, and then invasiveness [29]. The adhesion of cells, as well as their migration and MMPs secretion, is also involved in physiological processes in mammary gland tissue; thus, any alteration in this process might trigger significant consequences [30]. We observed that AOH significantly decreased the migration of 184A1 cells, simultaneously with decrease in *MMP-2* and *MMP-9* expression and activity, as well as modulation of the expression of *ZEB2* and *SNAIL* transcription factors involved in EMT process. To the best of our knowledge, this is the first study which reports that AOH might affect migration of normal breast cancer cells and modulate the expression of EMT-involved agents. Although AOH was reported to be the weaker estrogenic agent than 17β-estradiol, it might be crucial to understand its role in motility of both normal, as well as cancer, cells [14]. Akt signaling pathway regulates proliferation and cell cycle regulation. Its misregulation is commonly observed in breast cancer cells [31]. Similarly, MAPK is a highly conserved signaling pathway which regulates fundamental cell activities [32]. Previously, AOH was reported to modulate the expression of p38/MAPK in mouse embryonic NIH3T3 cells as a DNA damaging agent [33]. AOH was also reported to modulate inflammatory response via modulation of NFκB [16], in which activation simultaneously with MAPK signaling pathway might be an effect of proinflammatory stimulus [34]. No previous reports showed that AOH might modulate the Akt signaling pathway; however, another genotoxic mycotoxin (ochratoxin A) is known to activate both PI3K/Akt, as well as MAPK/ERK1-2, signaling pathways in HK-2 cells [35]. Similarly, another estrogenic mycotoxin zearalenone (ZEA) is reported to modulate these signaling pathways [36]. Thus, it is possible that AOH might also affect this two signaling pathways, but this statement needs further studies to be confirmed.

## 4. Materials and Methods

### 4.1. Chemicals

Alternariol (AOH) from *Alternaria* sp. (purity ~96%), estrogen (E2), dimethyl sulfoxide (DMSO), 4′,6-diamidine-2′-phenylindole dihydrochloride (DAPI), RIPA buffer, phenylmethylsulfonyl fluoride (PMSF), protease and phosphatase inhibitors, paraformaldehyde (PFA), acetic acid, methanol, ethanol, crystal violet, Triton X-100, and Coomassie brilliant blue were derived from Sigma Aldrich (St. Louis, MO, USA). Cell culture supplements: Dulbecco’s Modified Eagle Medium (DMEM), fetal bovine serum (FBS), HEPES buffer, sodium pyruvate, L-glutamine, and penicillin-streptomycin-neomycin solution (PSN) were derived from Thermo Fisher Scientific (Waltham, MA, USA). AlamarBlue^®^ reagent, TRIzol reagent, Geltrex™ hESC-Qualified Ready-To-Use, and Reduced Growth Factor Basement Membrane Matrix were also derived from Thermo Fisher Scientific (Waltham, MA, USA).

### 4.2. Cell Culture

Mammary gland epithelial cells 184A1 were derived from American Type Culture Collection (LGC Standards, Middlesex, UK) and cultured in DMEM supplemented with 10% heat inactivated FBS, 1% of HEPES buffer, sodium pyruvate, L-glutamine, and PSN. DMEM without phenol red, FBS, and PSN was used as experimental medium. Cells were used between passages 2 and 20.

AOH was dissolved in DMSO as stock solution of 0.01 M and freshly dissolved in experimental medium before usage. The concentration of DMSO in the used AOH highest concentration (10 µM) was lower than 0.1% and did not affect cells viability. Thus, for all experiments, cells treated with experimental medium were used as control. E2 in the concentration of 10 nM was used as positive control.

### 4.3. Cell Viability

AlamarBlue^®^ reagent was used to determine viability of cells. Briefly, 12 × 10^4^ of cells were seeded onto 96-well plates. The next day, the cultured medium was exchanged for experimental medium. Cells were treated with AOH in a concentration range 100–0.001 µM for 24 and 48 h, the doses of AOH were chosen on the basis of previous studies [10]. IC_50_ (the half maximal inhibitory concentration) was calculated for 24-h treatment (GraphPad Prism software, San Diego, CA, USA). Based on IC_50_ value and reported concentration of AOH in food samples [21], two concentrations of AOH were chosen for the rest of experiments: high (10 µM) and low (0.1 µM). In all experiments, cells were treated with AOH for 24 h. Morphology of cells was visualized with light microscope Olympus DP20 camera (magnitude 40×, Olympus Corporation, Tokyo, Japan).

### 4.4. Oxidative Stress and DNA Damage

An induction of oxidative stress caused by AOH was evaluated by counting ROS positive cells with Muse Oxidative Stress Kit (Merck Millipore, Burlington, MA, USA). The detection of ROS positive cells is based on dihydroethidium (DHE) reagent. DNA damage was detected with Muse Multicolor DNA damage Kit (Merck Millipore, Burlington, MA, USA) and expressed as p-ATM and pH2A.X positive cells. For those experiments, cells in the number of 0.6 × 10^6^ were seeded onto 6-well plates and treated with AOH for 24 h. The procedure of staining and counting was conducted according to the manufacturer’s recommendations. The experiment was conducted in triplicate. Fragmentation of DNA was visualized with DAPI staining.

### 4.5. Cell Cycle Analysis

Distribution of cell cycle was determined with Muse Cell Cycle Kit (Merck Millipore, Burlington, MA, USA) according to the manufacturer’s recommendations. The staining is based on propidium iodine (PI) incorporation to DNA. Cells were seeded and induced as described above. The experiment was conducted in triplicate.

### 4.6. Real Time Quantitative Chain Reaction (RTqPCR)

Cells (1 × 10^6^) for RNA isolation were seeded onto 60-mm Petri dishes. RNA was isolated with TRIzol. The concentration and purity of RNA was determined spectrophotometrically (Bio Drop DUO, BioDrop, Cambridge, UK). Five micrograms of RNA was used for cDNA synthesis with ImProm RT-IITM reverse transcriptase according to the manufacturer’s recommendations (Promega, Madison, WI, USA). RTqPCR was conducted on Roche 96 Light cycler (Roche, Basel, Switzerland) with 2 µL of cDNA. Primers were designed with Prime-BLAST software (National Institute of Health, NIH, Bethesda, MD, USA) (Table 1). Human Reference RNA (Stratagene, La Jolla, CA, USA) was used as calibrator for each reaction. The relative expression of genes was calculated with ΔΔCt method. As reference genes were used ribosomal protein S17 (*RPS17*), ribosomal protein P0 (*RPLP0*), and histone H3.3A (*H3F3A*). Each experiment was conducted in duplicate of three independent experiments and expressed as a relative expression.

### 4.7. Western Blot

Western blots was used to evaluate the expression of proteins. Cells (2 × 10^6^) were seeded onto 100-mm Petri dishes and cultured until reached 90% of confluence. Then, cells were induced as described above. After 24 h, cells were scratched, and protein was isolated with RIPA buffer supplemented with phosphatase and protease inhibitors, as well as PMSF. The concentration of protein was determined with Direct Detect^®^ (Merck Millipore, Burlington, MA, USA). The probes of three replicates were mixed together, and 30 µg of protein was used for electrophoresis and then transferred onto poly vinylidene fluoride (PVDF, Sigma Aldrich, St. Louis, MO, USA) membranes with wet transfer (400 mA, 110 min). Primary antibodies (Cell Signaling Technology, Leiden, Holland) were used: E-kadherin #3195, vimentin #5741, Akt #9272, phospho-Akt (Ser473) #4060, p44/42 #4695, phospho-p44/42 (Thr2020) #4370, cleaved-PARP1 (Asp214) #5625, SOD1 #4266, SOD2 #13141. GAPDH antibody (sc-32233, Santa Cruz Biotechnology, Heidelberg, Germany) was used as reference. Secondary antibodies conjugated with alkaline phosphatase (Sigma Aldrich) were used. Densitometry analysis was conducted in ImageJ program (ImageJ software, https://imagej.nih.gov/ij/, NIH).

### 4.8. Monolayer Wound Migration Assay (Scratch Assay)

Motility of cells is associated with its migration and invasion, as well as adhesion, to extracellular matrix proteins (ECM). Thus, we decided to evaluate if AOH might modulate this process. A migration of cells was evaluated with scratch assay. Cells were seeded onto 24- well plates and cultured until reach 100% of confluence. Then, a scratch was done with 100 µL tip, the wells were washed with phosphate-buffered saline (PBS), and experimental medium was added for 24 h. Scratches were photographed at time 0 and after 24 h with an Olympus CKX41 microscope (Olympus) with an Olympus DP20 digital camera (Olympus). The wound closure was calculated as a difference between the scratched area after 0 and 24 h and expressed as % of control (non-treated cells). The experiment was conducted in triplicate.

### 4.9. Modified Boyden Chamber Assay

The ability of cells to invade was evaluated by modified Boyden chamber assay with cell culture inserts (polyethylene terephthalate (PET), 0.8 µm pores) coated with a thin layer of Geltrex™. Briefly, cells (1.5 × 10^5^) were seeded into the upper chamber of insert in experimental medium containing AOH (600 µL), whereas the lower chamber was filled with 2 mL of experimental medium supplemented with 10% of FBS to induce cells migration. Inserts were cultured in standard conditions for 24 h. Then, both inserts and companion plates were fixed with 4% PFA in PBS and stained with crystal violet. Cells from the upper side of insert were removed with cotton bud. Inserts and plates were dried and photographed, and stained cells were dissolved with 10% acetic acid. Absorbance was measured with a BioTek Plate Reader EL808IU (Biotek, Winooski, VT, USA) at 550 nm. The experiment was conducted in triplicate.

### 4.10. Cells Adhesion Assay

Cells adhesion to ECM proteins was evaluated with 24-wells plates coated with collagen I, collagen IV, laminin, and fibronectin (Corning, Corning, NY, USA). Briefly, cells were pretreated with AOH for 24 h and then trypsinized and seeded onto ECM coated plates (1 × 10^5^ per well) in experimental medium for an additional 1.5 h. Then, cells were washed with PBS twice, stained with crystal violet, and washed once again with distilled water to remove excessive staining. Next, the wells were dried and dissolved similarly to inserts. The absorbance was measured at 550 nm with a BioTek plate reader. The experiment was conducted in three independent replicates.

### 4.11. Gelatin Zymography

The activity of MMP-2 and MMP-9 was evaluated with zymography assay. Briefly, cells were seeded on 6-well plates and induced as described above. A protein concentration in medium after 24 h incubation was evaluated with Invitrogen QubitR Fluorometer (Thermo Fisher Scientific, Waltham, MA, USA). Five micrograms of protein was separated on 10% gelatin zymography gels (120 V, on ice) and then incubated in 2.5% Triton X-100 solution twice for 30 min. Next, gels were incubated in developing buffer for 48 h in 37 °C and then stained with Coomassie brilliant blue solution and destained in methanol: acetic acid (50:20) solution to visualize white bands. Gels were scanned, and the intensity of bands was calculated in ImageJ software (ImageJ). The experiment was run in triplicate.

### 4.12. Statistical Analysis

All results are expressed as mean ± SE. One-way ANOVA with Bonferroni post hoc test was used to calculate the significance of the results (GraphPad Prism software). *p* value lower than 0.05 was considered as statistically significant.

## 5. Conclusions

AOH was considered a not significant mycotoxin, due to the low number of studies and not fully elucidated mechanism of its bio-distribution in animals [10]. However, today, more and more studies are focused on determining the mechanism of action of AOH in mammals, based on the studies indicating that AOH might act as estrogenic and immunomodulatory agent [12,14] and potentially affect human health. Thus, revealing the mechanism of action of AOH is crucial to determine the consequences of AOH exposure in the environment. This study, for the first time, showed that potentially estrogenic mycotoxin AOH might affect viability and motility of normal mammary gland epithelial cells via induction of oxidative stress, DNA damage, and cell cycle modulation and might constitute an issue in further research concerning the effect of mycotoxins on human health.

## Figures and Tables

**Table 1:** The relative expression of Akt and p44/42 and their phosphorylated forms obtained in Western blot analysis. The results are expressed as a fold change as compared to reference GAPDH expression calculated in ImageJ as integrated optical density. Akt–protein kinase B, p44/42–Erk1, AOH–alternariol, E2–estradiol 10 nM, Cnt–control.

Treatment	Relative Protein Expression			
	Akt	p44/42	Phospho-Akt	Phospho-p44/42
10 µM AOH	1.2287	1.2250	2.2522	1.5986
0.1 µM AOH	1.3859	1.3199	2.4724	1.7217
E2	1.4221	1.4095	2.5177	1.7714
Cnt	1.4696	1.4487	2.4906	1.9994

## Data Availability

Most of the data are presented in the study. The data not presented in this study are available on request from the corresponding author.
